# Identifying functional reorganization of spelling networks: an individual peak probability comparison approach

**DOI:** 10.3389/fpsyg.2013.00964

**Published:** 2013-12-25

**Authors:** Jeremy J. Purcell, Brenda Rapp

**Affiliations:** Department of Cognitive Science, Johns Hopkins UniversityBaltimore, MD, USA

**Keywords:** spelling, dysgraphia, ALE, IPPC, fMRI, orthography, mahalanobis

## Abstract

Previous research has shown that damage to the neural substrates of orthographic processing can lead to functional reorganization during reading (Tsapkini et al., 2011); in this research we ask if the same is true for spelling. To examine the functional reorganization of spelling networks we present a novel three-stage Individual Peak Probability Comparison (IPPC) analysis approach for comparing the activation patterns obtained during fMRI of spelling in a single brain-damaged individual with dysgraphia to those obtained in a set of non-impaired control participants. The first analysis stage characterizes the convergence in activations across non-impaired control participants by applying a technique typically used for characterizing activations across studies: Activation Likelihood Estimate (ALE) (Turkeltaub et al., 2002). This method was used to identify locations that have a high likelihood of yielding activation peaks in the non-impaired participants. The second stage provides a characterization of the degree to which the brain-damaged individual's activations correspond to the group pattern identified in Stage 1. This involves performing a Mahalanobis distance statistics analysis (Tsapkini et al., 2011) that compares each of a control group's peak activation locations to the nearest peak generated by the brain-damaged individual. The third stage evaluates the extent to which the brain-damaged individual's peaks are atypical relative to the range of individual variation among the control participants. This IPPC analysis allows for a quantifiable, statistically sound method for comparing an individual's activation pattern to the patterns observed in a control group and, thus, provides a valuable tool for identifying functional reorganization in a brain-damaged individual with impaired spelling. Furthermore, this approach can be applied more generally to compare any individual's activation pattern with that of a set of other individuals.

## Introduction

In recent years there has been a growing interest in understanding the functional regions that are required to produce written language, i.e., spelling (for reviews see Purcell et al., 2011; Planton et al., 2013). Although understanding how spelling is instantiated in the brain in neurologically intact individuals is of fundamental importance, it provides a somewhat limited view of the flexibility and resilience of brain networks associated with spelling. A deeper understanding of the manner in which the brain instantiates an evolutionarily recent skill such as spelling would benefit from the study of individuals who have suffered damage to the network of regions associated with spelling, i.e., individuals with acquired dysgraphia. The study of such cases can shed light on the plasticity of the neural substrates that support written language processing and representation.

**Figure 1:**
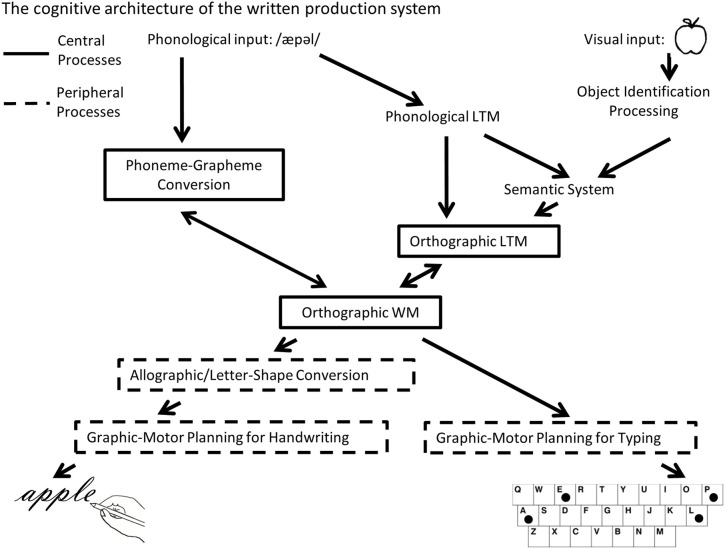
**A schematic depiction of the cognitive architecture of the written word production system that distinguishes between central and peripheral spelling processes (adapted from Purcell et al., 2011)**.

There is a long history in neurology associated with understanding how written language production is represented in the brain by examining individuals with acquired dysgraphia (e.g., Gordinier, 1903). Such work has helped to characterize a complex cognitive system that relies on numerous different inter-connected cognitive processes (Roeltgen and Heilman, 1985; Rapp and Caramazza, 1997; Rapcsak et al., 2002; Hillis and Rapp, 2004). One valuable distinction that has emerged from this extensive body of literature refers to the central and peripheral components of written production [see Figure 1, adapted from Purcell et al. (2011)]. As depicted in Figure 1, the central component processes of spelling are comprised of orthographic long-term memory (LTM), phoneme-grapheme (PG) conversion and orthographic working memory (the graphemic buffer). Access to Orthographic-LTM representations of words can be based either on the meaning of a word or on its phonological representation (Patterson, 1986). Entry into the central spelling system can also come directly from a phonological stimulus by way of PG conversion which involves the mapping of the sub-lexical sound units to plausible graphemic units; this allows for the spelling of unfamiliar words whose spellings have not been previously stored in orthographic LTM. The graphemic units that are retrieved from these two processes are considered to be abstract, without format-specific information (such as shape, font, or size information) (Rapp and Caramazza, 1997). These abstract graphemic units are then temporarily retained in orthographic working memory (WM) which maintains letter identity and order information prior to processing in the more peripheral written production system (Rapp and Kong, 2002; Kan et al., 2006).

The more peripheral component processes of written production translate the abstract graphemic information processed in orthographic WM to the motor plans and programs needed for spelling in a specific format (e.g., handwriting, typing or oral spelling). Although the peripheral components are valuable for understanding the full cognitive mechanisms associated with spelling, the research reported in this paper focuses primarily on the central spelling processes of spelling. These comprise the core processes important for not only written production, but for any task that requires knowledge of a word's spelling, e.g., oral spelling, performing a word search, or playing scrabble, etc.

In recent years the neural underpinnings of these central processes of spelling in neurologically intact individuals have been investigated in a number of different neuroimaging studies of spelling. This body of work was examined in two recent meta-analyses of spelling (Purcell et al., 2011; Planton et al., 2013). The Purcell et al. (2011) meta-analysis, in particular, focused on characterizing the brain regions associated with the central processes of spelling. This study reported that the most consistent areas of activation across studies involving central spelling processes were in the left fusiform gyrus, left inferior frontal gyrus, bilateral superior temporal gyrus, and left intraparietal sulcus. This work indicates that these areas play a significant role in the retrieval and/or assembly of the orthographic representations used in spelling.

Consistent with the conclusion that these areas are involved in the central processes of spelling are the findings of several recent fMRI studies examining both spelling and reading in the same groups of participants. These studies identified overlapping activations in both the left fusiform and inferior frontal gyrus (Purcell et al., 2011; Rapp and Dufor, 2011; Rapp and Lipka, 2011). The left mid-fusiform region in particular has been previously identified as important for orthographic processing in reading and has been termed the Visual Word Form Area (VWFA) (McCandliss et al., 2003; Cohen and Dehaene, 2004; Dehaene et al., 2005; Baker et al., 2007; Dehaene and Cohen, 2011). Additionally, studies of individuals with lesions to the left fusiform gyrus have confirmed that this area is necessary for normal spelling of words (Rapcsak and Beeson, 2004), and that a lesion to this area can lead to impairment in both spelling and reading (Philipose et al., 2007; Tsapkini and Rapp, 2010). This fMRI and lesion work implicating the same subregions of the fusiform and the IFG in both reading and spelling suggest that the function of these areas is one which is common to both reading and spelling and, therefore, likely involves the central processing components involved in accessing and storing abstract orthographic information.

As this brief review indicates, a network of neural regions consistently associated with spelling has been identified from neuroimaging and neuropsychological work, providing a valuable opportunity to examine how these functional networks reorganize or remain intact when there is damage to one part of the network. The value in identifying the changes that occur after a part of a network is damaged has relevance for treatment, for understanding the resiliency of the written language network and, more generally, for understanding how the brain responds to injury. In recent years an expanding body of research has focused on characterizing functional reorganization in the brain after neural injury to the left, language-dominant hemisphere (Price and Crinion, 2005; Crinion and Leff, 2007; Thompson and den Ouden, 2008). A current debate in the field regards the nature of the reorganization that leads to functional recovery. Some studies indicate that neural recovery is associated with a shift in language function to the right, or non-language dominant hemisphere (Weiller et al., 1995; Ohyama et al., 1996; Cappa et al., 1997; Musso et al., 1999; Thulborn et al., 1999; Xu et al., 2004), while others suggest that it is associated with heightened ipsilesional neural activity (Karbe et al., 1998; Cao et al., 1999; Warburton et al., 1999; Heiss and Thiel, 2006; Van Oers et al., 2010). Both patterns of reorganization may be involved as posited in work which suggests that whereas the presence or increase in contralesional activation tends to occur within the first year of a stroke, afterwards functional reorganization primarily involves recruitment of ipsilesional cortex (Saur et al., 2006; Saur and Hartwigsen, 2012). A recent meta-analysis of the literature further suggests that these two patterns may vary depending on the specific region of the language dominant hemisphere that has been damaged; Turkeltaub et al. (2011) found that individuals with left IFG lesions tended to engage contralesional IFG cortex, whereas lesions to non-IFG areas tended to be associated with ipsilesional patterns of activation.

Critical for this work is the question of how one detects functional reorganization in the brain. The methods used to examine reorganization have primarily focused on identifying a pattern of fMRI results in a control group and then comparing it in some way to data from lesioned individuals. Much of this work has focused on grouping the data from multiple lesioned individuals, averaging them and then performing a random effects analysis comparing the two groups (e.g., Blank et al., 2003; Crinion et al., 2006). Although there are benefits to such an approach, there is typically considerable variability in the activation location and behavioral symptoms across the lesioned individuals. Therefore, differences at the individual level in patterns of reorganization may be missed. Studies which do compare patterns of activation in an lesioned individual to the pattern exhibited by a control group primarily rely on qualitative comparisons. These qualitative comparisons either involve comparing the activation map from a single patient to the average activation map from a neurologically intact group (e.g., Warburton et al., 1999; Perani et al., 2003; Thompson et al., 2010), or involve comparing the activation map from a single patient to the activation map from a neurologically intact individual, (e.g., Postman-Caucheteux et al., 2010).

Another approach to studying reorganization is to examine activation patterns before and after treatment that targets specific components of a language function (e.g., for reviews and discussion of methodological issues, see Crinion et al., 2013; Kiran et al., 2013; Meinzer et al., 2013; Rapp et al., 2013). This approach aims to identify the neural changes associated with the specific language changes targeted by treatment (e.g., Fridriksson et al., 2012). A strength of this approach is that comparisons of pre and post-treatment activation patterns provide for a greater understanding of the functional significance of the observed patterns of reorganization that are observed.

Another, less common method involves pre and post-operative comparisons of the functional data in individuals undergoing a surgical resection. This method was employed in a seminal study in reading, reporting on the functional activation maps in an individual both before and after undergoing a resection of the left posterior fusiform gyrus (Gaillard et al., 2006). The fMRI results pre-operatively suggested that there was a relatively normal neural response to faces, objects and written stimuli (although no method of quantifying this normality was applied). Post-operatively it was revealed that the pre-operative normal response to written stimuli in the left fusiform was disrupted, and replaced with perilesional activation posterior to the location of the pre-operative activation.

Recent attempts to quantify the degree of functional abnormality in individuals with neurological damage have involved performing direct comparisons between the functional activation observed in an individual patient to the average functional activation in a group of control subjects. For instance in the work of Fridriksson et al. (2010) a set of patients with aphasia participated in an fMRI task of naming; activation associated with naming in each patient was compared to the average naming activation observed in a control group. This yielded a contrast parameter for each patient that was further examined in order to correlate naming behavior with the fMRI results in the set of patients. The logic was to characterize the fMRI map from the group as the “gold standard” of activation, and patient activation relative to this provides a measure of deviation from the norm (Fridriksson et al., 2010). Although this work provides a valuable method for examining the degree of difference of the individual subject to a control map functional activation map, it does not take into account the inherent variability of the responses within the control group in this comparison.

All of these approaches have specific strengths and weaknesses. A review of this work reveals that, to date, few studies have applied statistical methods to compare the pattern of activation from a single lesioned individual to that of a set of control individuals in a way that accounts for the often considerable variability in the activation patterns of the non-impaired individuals. Although methods for comparing data from an individual to that of a group of controls is available for behavioral studies (Crawford and Garthwaite, 2002), it is generally lacking in neuroimaging. These methods, however, are essential when studying individuals with acquired language disorders when they demonstrate heterogeneous behavioral profiles and distributions of neural damage. Therefore, methods that permit the comparison of one-to-many are highly valuable when characterizing the abnormality (or normality) of the activation profiles of individuals with acquired language disorders.

One recent study presented a novel method for comparing the functional response of a brain-damaged individual to the activation patterns from a set of control individuals in the domain of reading (Tsapkini et al., 2011). They applied a novel statistical approach based on the Mahalanobis distance analysis (Mahalanobis, 1936) that involved determining if the locations of activation peaks in the brain-damaged individual were abnormal relative to those of a set of individual controls participants. Here we introduce a novel approach that we refer to as the Individual Peak Probability Comparison (IPPC) analysis which builds on the Tsapkini et al. (2011) work. The objective of this analysis approach is to characterize the neuro-topography of functional activation. This is done by focusing on functional peak locations rather than on the magnitude or volume of functional activation clusters.

The IPPC analysis provides a method for comparing the activation patterns obtained during fMRI from a single brain-damaged individual to those obtained from a set of non-impaired control participants. Briefly, Stage 1 of the analysis characterizes the convergence in activations across a set of non-impaired control individuals by applying an analysis technique typically used for characterizing activations across studies: Activation Likelihood Estimate (ALE) (Turkeltaub et al., 2002). The results of this stage of the analysis correspond to a set of peak coordinates that have the highest likelihood of being active across the set of control subjects. This provides a method for identifying brain areas of consistent activation across a group of control subjects. Stage 2 provides a characterization of the degree to which the brain-damaged individual's activations do or do not correspond to the group pattern identified in Stage 1. This analysis involves using the Mahalanobis distance statistic (Mahalanobis, 1936) to compare each of the control group's peak activation locations to the nearest peak generated by a single brain-damaged individual (Tsapkini et al., 2011). The Mahalanobis distance (MD) is a scale-invariant distance measure that is sensitive to the covariance of the distribution of peak locations in the group and, therefore, is useful for determining the abnormality of peak functional activation locations in an individual. The aim of Stage 3 is to determine whether the brain-damaged individual's peak activation locations are typical/atypical relative to the range of variation in the peak activation locations observed in the control participants. This involves comparing the brain-damaged individual's peak activation locations with probabilistic-peak maps (PPMs) generated for each of the control participants. In this way it is possible to determine, for each of the brain-damaged individual's activation peaks, whether it is in a location that is commonly or rarely observed in the control population.

Together this three pronged analysis provides a valuable method for comparing the peak activation locations of an individual to those of a group of individuals via objective criteria and sound statistical methods. The findings from this type of analysis provide a useful tool for determining if the functional activation patterns in an individual fall outside of the normal range as compared to a group of controls; critically this determination takes into account the variability in the location of the activation patterns in the set of control participants.

In summary, surprisingly little research has focused on understanding functional reorganization in the brain in individuals with acquired language disorders, let alone acquired dysgraphia. Here we use fMRI to identify a typical pattern of functional activation associated with spelling in neurologically intact individuals and then apply a novel IPPC analysis method for comparing this typical spelling pattern to the spelling pattern identified in an individual with acquired dysgraphia.

### Case study

**Figure 2:**
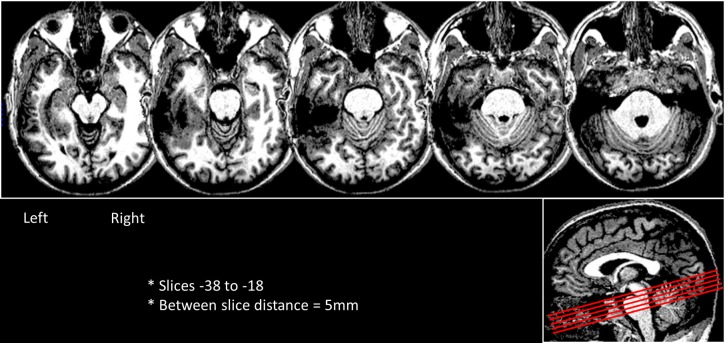
**Axial slices depicting DPT's lesion in the left ventral occipitotemporal cortex**. The slices were rotated -15 degrees from the AC-PC line and are shown in a sagittal view as red lines in the right side box.

DPT was a right-handed, lawyer by profession (DOB: 9/1969) who underwent surgical resection of an oligodendroglioma in the left fusiform gyrus (2001). Immediately after the surgery DPT experienced impairments in spoken naming, reading comprehension, spelling and short-term memory. In the first month after his surgery there was sufficient behavioral recovery from these deficits that he was able to return to practicing as an attorney and continued to work in this capacity throughout the time that the data for this study were acquired. Still, he continued to have mild difficulties in reading and moderate difficulties in spelling combined with modest anterograde memory deficits. His reading and spelling abilities prior to the surgery were considered to be normal or above normal based on the self-report that his work involved extensive reading and that his spelling was normal as compared to his fellow law-school graduates. The behavioral and structural and functional MRI data reported here were collected during the same period as the behavioral testing summarized below and reported in detail in (Tsapkini and Rapp, 2010). As shown in Figure 2, the resection lesion site primarily encompasses a large portion of the anterior and mid left fusiform gyrus and part of the medial portion of the anterior left inferior temporal gyrus. The lesion extent in the x, y, and z dimensions in MNI coordinates was as follows: along the medial–lateral axis from approximately *x* = −29 to −63, along the anterior-posterior axis from *y* = −15 to −66, and along the superior–inferior axis from *z* = −30 to −6.

### Behavioral testing

A clinical neuropsychological evaluation 21 months after the resection (6/2003) indicated that DPT demonstrated normal or superior performance in numerous cognitive areas including verbal WM, visual perception and memory, fine motor speed and precision, spoken word naming and fluency, oral reading, single word auditory comprehension, and recognition memory for words and face (for further details see Tsapkini et al., 2011). In addition, during the period of 7/2005–8/2007, DPT's behavioral performance was examined extensively with regards to: (1) orthographic processing (reading of words and pseudowords), visual lexical decision with semantic priming, written synonym judgments and written spelling of words and pseudowords; (2) auditory word processing (auditory lexical decision with semantic priming and auditory synonym judgments) and (3) visual object processing (for faces: fame and profession judgment tasks; for visual objects: spoken picture naming and object comprehension). DPT's performance was compared to that of 11 age- and education-matched control participants using a modified *t*-test (Crawford and Garthwaite, 2002). On the basis of this testing (summarized in Table 1), Tsapkini and Rapp (2010) concluded that DPT suffered from a deficit that was specific to the orthographic modality in that both comprehension (reading) and production (spelling) of words were impaired, while in the auditory modality both comprehension and spoken production of words were spared. Furthermore, the deficit for reading was considered specific to the visual category of written words because processing of pseudowords, faces and visual objects was unaffected (other categories of visual objects were not systematically evaluated). These behavioral tests provided the following findings with regards to DPT's behavioral profile: (1) normal processing and access to semantics for faces and visually presented objects and auditorily presented words; (2) sparing of sublexical (pseudoword) processing in both reading and spelling; and (3) disruption of lexical (word) processing in both reading and spelling, most likely affecting the translation between orthographic word forms and lexical semantics. Here, we carried out an fMRI study in order to specifically examine the neural response patterns associated with spelling so as to determine if DPT's abnormal behavioral pattern in spelling was reflected in the neural responses.

**Table 1:** **Summary of behavioral testing reported in Tsapkini and Rapp (2010) and Tsapkini et al. (2011)**.

**Task**	**DPT**	**Controls mean (range) (*SD*)**	**Statistical comparison DPT vs. controls**
**SPELLING**			
Words	81% (55/68)	96–100% (0–3/68)	*p* < 0.05
Pseudowords	97% (33/34)		
Pseudoword reading	759	582 (465–804) (117)	ns
**WORD READING**			
High frequency	583	470 (388–596) (62)	ns
Low frequency	615	477 (398–617) (65)	*p* < 0.1
Regular	588	475 (391–608) (64)	ns
Exception	632	477 (393–614) (63)	*p* < 0.05
**VISUAL LEXICAL DECISION**			
High frequency	675	572 (465–766) (91)	ns
Low frequency	807	640 (537–809) (84)	*p* < 0.1
Regular	718	602 (490–767) (89)	ns
Irregular	712	574 (535–801) (82)	ns
Strange	727	597 (484–767) (86)	ns
**SYNONYM JUDGMENT**			
Written	976	762 (564–965) (105)	*p* < 0.1
Auditory	1382	1036 (719–1456) (248)	ns
**VISUAL SEMANTIC PRIMING**			
Overall lexical decision	698	633 (486–880) (128)	ns
Priming effect: unrelated-related	12	43 (29–73) (18)	*p* < 0.05
**AUDITORY SEMANTIC PRIMING**			
Overall lexical decision	1183	1101 (964–1363; 150)	ns
Priming effect: unrelated–related	169	200 (152–283; 46)	ns
**SPOKEN PICTURE NAMING**			
High frequency	822	687 (579–881; 116)	ns
Low frequency	1035	788 (683–932; 128)	ns
High complexity	1098	806 (681–915; 122)	ns
Low complexity	1140	817 (654–1059; 164)	ns
Faces: fame judgment	1183	938 (772–1434; 235)	ns
Faces: occupation categorization	838	823 (523–1123; 163)	ns
Object comprehension	2269	1765 (1147–2565; 504)	ns

## Materials and methods

### Overview

In order to compare DPT's functional activation patterns to those of the set of control participants, we performed two sets of analyses. The first was a simple random-effects analysis for the two sets of control participants and a fixed-effects, single-subject analysis for DPT. These analyses allowed for us to present whole-brain functional activation maps associated with spelling from DPT and the control groups. Second, we performed a novel multi-step IPPC analysis. This allowed for the comparison of the location of DPT's activation peaks to those of the control participants by taking a probabilistic approach to peak activation location that relies on both meta-analytic and MD analysis methods.

### Subjects

There were two groups of control participants, for a total of 19 control participants. Group 1 included 10 individuals (4 male) with an age range from 18 to 42 and an education level of some college education or higher. Group 2 included 9 individuals (8 male) with an age range from 30 to 42 and an education level of a B.A. degree or higher. All individuals in both groups were right-handed according to the Edinburgh Inventory (Oldfield, 1971) with no history of neurological impairment and no known history of reading or spelling deficits. Each participant was a native English speaker (English was their first language) and had no detectable spelling deficits as determined by a short spelling test administered prior to the scanning session. Each participant was recruited from the Johns Hopkins University community, provided written informed consent that was approved by Johns Hopkins University Institutional Review Board, and was paid for their participation.

### Experimental tasks

Our main goal was to examine, with a spelling task, the differences in the locations of brain activations between DPT and the set of control participants. We administered an fMRI block design spelling experiment to the two sets of control groups. There were two task conditions: a spelling task and a control case-verification task; there was also a fixation rest period between blocks. Both the spelling and the case verification tasks involved the same sensory and motor components and only varied on the instructions that were given for each task. For the spelling task, each trial was 6 s. and proceeded as follows: (1) a 1500 ms centrally displayed task prompt (SPELLING?); (2) a 300 ms central fixation cross, (3) 1200 ms time for auditory word presentation with variable initial silence period, (4) a 1000 ms visually displayed uppercase letter, and (5) a 2000 ms blank screen to allow for a button response. Participants were instructed to press a button if the visually presented letter was contained within the spelling of the auditory word (right hand if yes/left hand if no). The case-verification task was the same except that the task prompt was UPPER-CASE? the visually displayed letter could be presented in either upper- or lowercase, and participants were instructed to respond yes/no (button press) whether the visually presented letter was or was not in uppercase; participants were further informed that the auditorily presented word was irrelevant to this task.

The tasks were designed so that a comparison of the spelling task to the case verification task would identify the central components of the spelling process without the need for writing in the scanner. The rationale was that both tasks involve similar auditory input, visual stimuli (task cue and the letter probe), and motor responses; therefore the case-verification task controls for these components of the trial when compared to the spelling task trials. For the spelling task, participants must access orthographic LTM (and/or sub-lexical spelling processes) and then engage orthographic WM while identifying if the probe letter corresponds to a letter in the word spelling; these processes are not required in the case verification task. Therefore, when compared to the case verification trials, central component processing of spelling (orthographic long term memory, sub-lexical processes, and orthographic long term memory) will be selectively activated. This task and the same set of subjects in Group 1 was reported in Rapp and Lipka (2011). The findings reported by Rapp and Lipka (2011) were highly similar to the “normal spelling network” associated with the central component processes as described by a recent meta-analysis of spelling (Purcell et al., 2011; see Figure 1).

There were some differences between how the spelling experiment was designed for Group 1 and Group 2 which motivated some differences in data analysis. For Group 1, each block contained 6 trials (36 s per blocks) with 6 s. of fixation rest between blocks; the experiment was presented in 6 runs, each with a scan duration of 264 s. acquisition time per run. For Group 2 each block contained 9 trials (48 s per block) with 6 s. of fixation rest between blocks; the experiment was presented in 4 runs, each with a scan duration of 372 s acquisition time per run. Additionally, for Group 1, different auditory words were presented on every trial for both the spelling and case-verification tasks, while for Group 2, in the case-verification task only, a small set of auditory words were used and repeated in every block of the task. DPT participated in the same experimental design as did Group 2. Two fMRI data sets were obtained for DPT on different days.

### Imaging parameters

MRI data collection for Group 1 was carried out with a 1.5 T Phillips scanner and for Group 2 and DPT a 3T Phillips scanner was used. For both groups, T2^*^-weighted fMRI signals were measured using a gradient echo, echo-planar imaging sequence with the following specifications. Although similar, spelling experiments were administered to both Group 1 and Group 2, there were slight differences in the acquisition timing for each group. The scan parameters for Group 1 were as follows: repetition time *TR* = 1500 ms, *FA* = 65°, *TE* = 30 ms, *FOV* = 240 × 240 mm, matrix = 128 × 128; 176 brain volumes were collected with 29 interleaved axial slices and a 4 mm slice thickness. The scan parameters for Group 2 were as follows: repetition time *TR* = 1500 ms, *FA* = 70, *TE* = 40 ms, *FOV* = 230 × 230 mm, matrix = 64 × 64; 248 brain volumes were collected with 23 sequential axial slices and a 5 mm slice thickness. Comparable, full-brain coverage was obtained in both Group 1 and 2. High resolution MP-RAGE T1-weighted scans (1-mm isotropic voxel resolution) were acquired for each participant from both Group 1 and 2 as well as for DPT. Slightly different structural imaging parameters were used for each group; these scans were used for co-registration and normalization to the Talairach and Tournoux atlas (1988). For Group 1 the following parameters were used: *TR* = 8.06 ms, *TE* = 3.8 ms, matrix = 256 × 256, *FOV* = 256 × 200, and 200 slices with 1 mm thickness. For Group 2 and DPT the following parameters were used: *TR* = 8.28 ms, *TE* = 3.8 ms, flip angle = 8°, matrix = 256 × 256, *FOV* = 256 × 180, and 200 slices with 1 mm thickness.

### fMRI data analysis

Functional and anatomical data were analyzed using Brain Voyager QX 2.4 (Brain Innovation, Maastricht, The Netherlands) and Matlab (The Math Works). Functional scans were preprocessed with the following sequential steps: motion correction, inter-slice acquisition time correction, temporal high-pass filtering (3 cycles per time series), functional-anatomical co-registration, and normalization to Talairach space.

#### Analysis 1: whole-brain comparison of DPT with controls

***Control participants data analysis***. Both Group 1 and Group 2 control groups were subjected to separate whole-brain random-effects analyses. For both sets of control groups, functional data were smoothed with an 8 mm full-width half-maximum Gaussian kernel. We convolved a canonical hemodynamic response function with the time points associated with the conditions of interest and then employed a general linear model approach to estimate the parameters associated with these conditions. For the data from Group 1 we employed the same design model that was used to analyze these data in a previous study (Rapp and Lipka, 2011). Briefly, for Group 1, a block design was applied in which the time periods corresponding to the spelling task block and the fixation period were explicitly modeled, whereas the case-verification task time points were left un-modeled and served as the implicit baseline condition. The critical comparison for isolating the spelling network was between the spelling blocks and the case-verification baseline condition. For Group 2 we used an event-related model with regressors corresponding to the following components of the trial: the initial task-cue, auditory word, visual letter, and silent inter-trial-interval period. These regressor types were applied to both the spelling probe and the case-verification task trials. The critical contrast used for isolating the spelling network was between the auditory word presentation plus the visual letter presentation portion of the spelling probe trials as compared to the corresponding portion of the case-verification trials; in the spelling trials this is the time period during which participants must mentally generate a word spelling and evaluate it for the presence/absence of the target letter.

For both Group 1 and Group 2 analyses serial correlations were accounted for by incorporating a standard AR1 auto-regressive correction method. In order to account for non-neuronal physiologically induced signal variations for noise we included the average signal at each timepoint within the cerebral spinal fluid (CSF) as regressors of no interest (Birn et al., 2006).

We then subjected the data from Group 1 and Group 2 to random effects analysis. This analysis was focused on the comparisons of the aforementioned critical contrasts used to isolate spelling. An initial voxel-wise threshold of *p* < 0.02 was applied; corrected significance for clusters of activation was determined by using cluster size thresholding via a plug-in implemented in BrainVoyager: a corrected *p* value of 0.05 was used (Forman et al., 1995). Both maximum and local-maximal peaks (also referred to as subpeaks) were reported. Local-maximal peaks were identified via a Brainvoyager compatible NeuroElf toolbox which applies a watershed search method that identifies each cluster peak and then uses a search algorithm that follows the steepest descent in order to delineate between local-maximum peaks. All coordinates reported herein are in Talaraich coordinate space (Talairach and Tournoux, 1988). Data are projected on to a standard template brain and rendered using MRIcron (Rorden and Brett, 2000).

***DPT's data analysis***. DPT's data were analyzed in the same manner as for Group 2, with the following differences. The data were analyzed with a fixed-effects regression model and considering we had two different sets of data for DPT from two different scanning sessions, we combined the data from both of the scan sessions. In order to allow for a comparison with the control group, DPT's data were smoothed at a FWHM of 8 mm. A voxel-wise threshold of 0.02 was applied and data were corrected at a *p* value of 0.05 via the same Monte Carlo simulation method referenced above.

#### Analysis 2: IPPC analysis

The IPPC analysis approach permits the statistical comparison of the activation patterns observed in DPT to that of the control groups. For this analysis we focus on the location of cluster activation peaks as opposed to cluster volumes. Although activation cluster volumes are valuable when characterizing individual and group activation maps, they are not ideal for applying a direct statistical comparison of individual activation maps to group activation maps. This is primarily because volumes are highly sensitive to thresholding levels and also demonstrate a considerable amount of variability in location even across neurologically intact individuals. The IPPC analysis, on the other hand, attempts to capture this inherent variability in functional responses by characterizing, in a probabilistic manner, the distribution of functional peak locations. This allows for a statistical comparison which determines if a peak from an individual functional map is an outlier when compared to the distribution of peak locations obtained from a control group. The IPPC analysis was carried out via the following 3 stage procedure.

***IPPC analysis stage 1***. The goal of this analysis stage was to characterize the “normal neural response pattern to spelling.” To do so, individual control subjects' data were first subjected to a fixed-effects analysis as described above for DPT (except that unsmoothed data were used). As for DPT, both peaks and local-maximal peaks were identified for each control participant. The significant positive maximal and local-maximal peaks from the spelling contrasts of interests in the control subjects served as input in a subject-based ALE analysis (Turkeltaub et al., 2002; Eickhoff et al., 2009). Although this method is typically applied to data from different studies, here we utilized individual subject data, treating each subject as an *n* = 1 study. This allows for a straightforward method of identifying regions of consistently high probability of activation peaks across individuals. The ALE method is described well in (Eickhoff et al., 2009); briefly, the purpose is to calculate the likelihood that a voxel in a normalized brain corresponds to a significant peak in a task/contrast of interest. This method relies on the premise that there is uncertainty regarding the precise location of each peak and that this uncertainty (which also takes into account the number of subjects in a study) can be modeled by the application of a 3-dimensional Gaussian probability density distribution. In our analysis, these Gaussian probability density distributions were applied to each peak for each subject. Once applied to each participant's activation peaks, the resulting individual probability maps were combined (summed) across participants in order to determine the ALE value in each voxel. High ALE values for a voxel correspond to high estimates of the likelihood that the voxel corresponds to a peak of activation in the set of participants. The statistical significance of these ALE values is determined by identifying the chance probability of finding each ALE value given the distribution of ALE values observed across the entire brain. A corrected *p* value was obtained with a false discovery rate (FDR) statistic. This analysis yielded “clusters” of significant ALE values that represent the brain locations which were most likely to include activation peaks, given the data from the contributing subjects.

***IPPC analysis stage 2***. The goal of this analysis stage was to address the question: To what extent does DPT's activation pattern correspond to the normal group. This was addressed by comparing DPT's activation to the normal spelling network identified in Stage 1. Specifically, we did so by determining the degree to which DPT's peak and subpeak activations were situated within the range of the control participant variability surrounding the peak locations in the normal network (which we refer to as ALE peaks).

First, for each of the ALE peak locations identified in Stage 1, we identified the participants that were the primary contributors to the high ALEs of that peak. To do so we identified participants with activation peaks within 2SD of localization uncertainty (see Eickhoff et al., 2009); for the *n* = 1 subject-based ALE analysis that we implemented this distance corresponds to 16.2 mm^1^]. This method has been applied previously in a meta-analysis to determine which studies contribute the most to each ALE peak (see Purcell et al., 2011). Each participant can only contribute a single peak to each set of peaks that are analyzed; if multiple peaks are initially identified for a single participant, only the peak with the closest Euclidean distance to the ALE peak location is included in the set. Having tallied the number of participants that significantly contributed to each significant ALE peak, we then excluded from further analysis ALE peaks which had which fewer than 4 contributors. We then characterized each of the remaining ALE peaks as either “strong” if a majority of the control participants contributed significantly to it or “weak” if fewer than half of the control participants contributed significantly to it. This distinction served to distinguish between the ALE peaks that represented the most consistent, (i.e., strong), normal neural response for spelling and those that, although significant in the ALE analysis, were less reliably recruited across participants (i.e., weak).

Next, for each ALE peak identified to be the strong normal neural response for spelling we identified the nearest DPT peak based on MD^2^. Mahalonobis distance was used, as opposed to Euclidean distance, because it considers the distance variability along each of the x, y, z dimensions separately (see Tsapkini et al., 2011 for more details), taking into account different degrees of variability along the different dimensions in the control data. We then calculated the MD of DPT's peak from the center of the distribution of the individual participant peaks that contributed to the ALE peak location. For each of these comparisons, the Mahalonobis distance values were then tested for significance against the critical value of the chi-square distribution with three degrees of freedom: χ^2^(3) = 7.81 for *p* < 0.05 (95% percentile). We generated figures depicting some of these comparisons, by plotting the x, y, z locations of the set of peaks from control participants and overlaying them with an ellipsoid centered on c (see footnote 2) that encompassed the 95% confidence interval. We used in-house programs developed in Matlab (The MathWorks, 2012) to perform this analysis and generate these plots.

Next, each DPT peaks was compared to the set of control participant peaks associated with the nearest ALE peak based on the MD. Here we want to determine *for each DPT peak* whether the nearest ALE peak is at a significant MD. This analysis included both strong and weak ALE peaks. This allowed us to determine if any of DPT's peaks were in atypical locations in relation to the strong and weak normal neural response pattern of spelling. A Chi-square test was applied to each of these MDs and a *p*-value was reported.

This stage allows for a characterization of whether DPT's peaks are normal, but does not address how abnormal the remaining DPT peaks are; in stage 3 this issue will be addressed.

***IPPC analysis stage 3***. By definition the peaks not characterized as normal in stage 2, can be considered abnormal. In this stage the typicality of these abnormal peaks will be characterized.

In order to examine the typicality of the location of each of DPT's peaks we generated what we will refer to as a PPM for the control participants. This was carried out by applying spheres around every control participant peak; each sphere had a radius equal to the 1 standard deviation of the UD (8.1 mm). This takes into account the uncertainty associated with each peak location. This map provided a method for visualizing, in a probabilistic manner, the regions in the brain that were more typically activated across a set of controls. Importantly, this type of map gives equal weight to every peak that is observed in the control data set and therefore permits visualization of the typical variability of the control participants in terms of peak-probability locations. Each of the DPT peaks was superimposed onto this map and the percentage of control participants that demonstrated a peak that overlapped with each DPT peak was calculated.

## Results

### fMRI results

#### Analysis 1: whole-brain comparison

**Figure 3:**
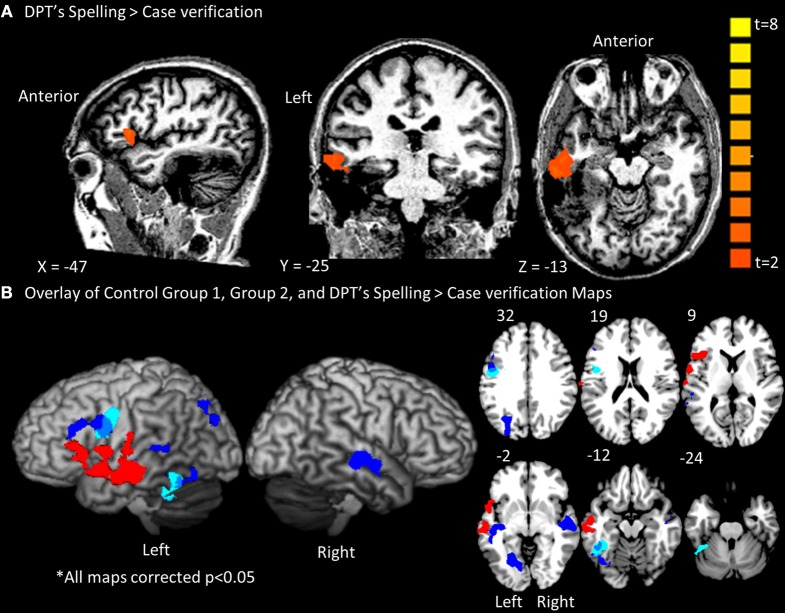
**Whole brain contrast maps depicting spelling activations for DPT and the control groups**. Only clusters surpassing a corrected cluster-threshold of *p* < 0.05 are shown. **(A)** Map of clusters with *t*-value scale for the Spell>Case Verification for DPT **(B)** Map of DPT's spelling clusters projected in red onto a standard rendered template brain and onto slices from 32 to −24 in the *z*-axis. Displayed also are the overlaid results from control groups 1(light blue) and 2 (dark blue) with blue-gray depicting the overlap between them. As can be seen, there was no overlap between the distribution of activations for control groups 1/2 and DPT.

***Group fMRI results***. As expected, the group-level random effects analysis yielded significant results in both control Group 1 and Group 2 (see Figure 3B). As reported in Table 2, the spelling > case-verification data from Group 1 demonstrated significant clusters in both the left fusiform gyrus which extended medially into the parahippocampal gyrus as well as in the left inferior frontal gyrus. The latter was located at the intersection of the Brodmann's Area 44, 9, and 6 near the inferior frontal sulcus which divides the inferior and middle frontal gyrus. This area has been termed the inferior frontal junction (Brass et al., 2005; Derrfuss et al., 2005). Also, as reported in Table 2, the spelling > case-verification data from Group 2 revealed activations in both of these areas as well. As shown in Figure 3B there is overlap of functional clusters in these two regions across both control groups. In addition, Group 2 also demonstrated activation in the following neuro-anatomical regions: left middle temporal gyrus, superior temporal gyrus, lingual gyrus, and a large cluster in precuneus that extended into the cuneus and the posterior intraparietal sulcus. This more extensive activation associated with Group 2 in relation to Group 1 is likely due to the difference in designs: whereas for Group 1 the control case-verification condition relied on different word stimuli in each run, in Group 2, these items were repeated, thereby providing a more “lenient” control condition which was more likely to detecting differences when compared to the spelling condition. These data also align with a previous meta-analysis which summarizes the consistent findings across multiple fMRI studies of spelling see (Purcell et al., 2011).

**Table 2:** **Random effects results from group 1 and group 2**.

**Location**		**Brodmann's area**	**Volume (mm^3^)**	**Peak (Max & local-max)**			**Max *Z*-value**
				***x***	***y***	***z***	
**GROUP 1**							
Left	IFG	9	4158	−45	−1	25	5.42
	IFG	44		−51	8	22	3.63
	PreCG	6		−27	2	28	4.08
Left	FG	37	3888	−45	−52	−14	9.36
	PhG	37		−33	−43	−8	6.83
**GROUP 2**							
Left	IFG	9	2835	−48	5	31	7.55
	MFG	9		−45	26	28	4.75
Left	FG	37	8424	−42	−55	−14	3.48
	FG	37		−42	−43	−11	4.53
	FG	37		−54	−55	−17	4.11
	PhG	19		−24	−58	−2	4.76
	PhG	28		−24	−22	−5	4.07
	MTG	21		−48	−31	1	5.33
	STG	41		−51	−37	13	5.13
	STG	22		−63	−43	13	3.99
	Lingual	19		−21	−70	−5	4.40
	Putamen			−33	−19	−2	7.33
Left	Precuneus	39	3402	−30	−64	31	5.96
	Precuneus	7		−18	−70	37	3.79
	SPL	7		−30	−73	43	4.54
	Cuneus	19		−27	−76	28	5.11

Anatomical Label: IFG, inferior frontal gyrus; MFG, middle frontal gyrus; FG, fusiform gyrus; PhG, parahippocampal gyrus; MTG, middle temporal gyrus; STG, superior temporal gyrus; SPL, superior parietal lobe.

***fMRI results for DPT***. As reported in Figure 3A; Table 3, DPT demonstrated activation in a single large cluster that included areas of the left inferior frontal gyrus and left temporal lobe. In the temporal lobe this cluster included portions of the anterior superior and middle temporal gyri. In the frontal cortex there was activation in the left inferior frontal gyrus in BA 44/45/6.

**Table 3:** **Significantly active regions for word spelling for DPT**.

**Location**		**BA**	**Size (mm^3^)**	**Peak (Max & local-max)**			**Max *Z*-value**
				***x***	***y***	***z***	
Left	TP	38	10584	−42	14	−23	2.34
	STG	22		−60	−10	4	2.24
	STG	22		−51	−7	−11	2.92
	STG	22		−51	11	−5	2.83
	^*^MTG	21		−60	−19	−8	3.62
	^*^MTG	21		−48	−22	−11	2.40
	IFG	45		−45	20	10	3.53
	IFG	45		−54	26	13	2.67
	IFG	44		−57	11	13	2.15
	PreCG	6		−54	2	10	2.79

* Perilesional peaks (within 5 mm of lesion space); Anatomical labels: TP, temporal pole; STG, superior temporal gyrus; MTG, middle temporal gyrus; IFG, inferior frontal gyrus; PreCG, precentral gyrus.

As indicated in Figure 3B, after overlaying DPT's results with those of Groups 1 and 2, there were no overlapping voxels. Given an outcome such as this one, it is particularly important to be able to quantitatively evaluate whether any of DPT's activation peaks, although not overlapping with those of the control group results, fall within normal range, considering the range of individual control participant variability. The IPPC analysis allows for just this determination.

#### Analysis 2: IPPC

**Figure 4:**
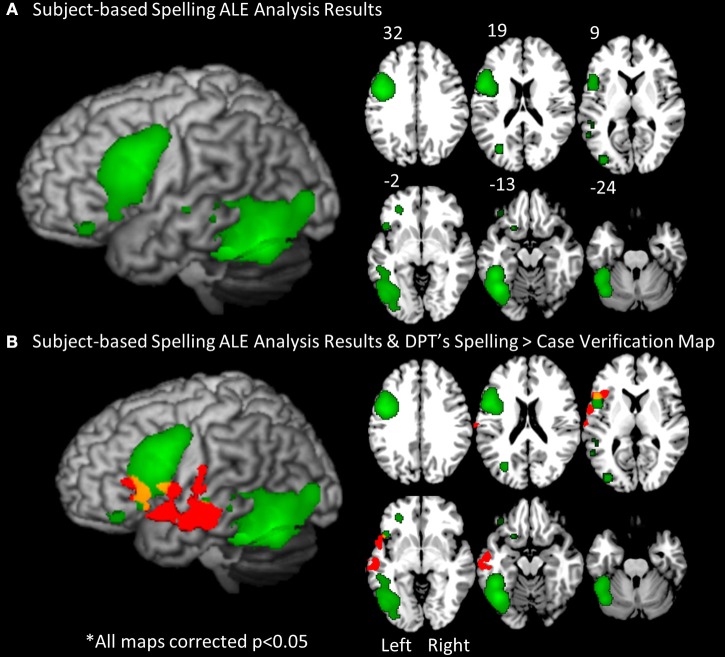
**The subject-based ALE analysis map**. **(A)** The results of the subject-based ALE analysis based on the locations of significant spelling peaks from every control participant (19 total). ALE clusters were FDR corrected at *p* < 0.05. Significant clusters were projected on a standard rendered template brain in green. These clusters correspond to the regions with greatest likelihood of peak activation for spelling across all control subjects. **(B)** An overlay of DPT's functional activation map in red (from Figure 3) and the subject-based ALE map in green. Areas of overlap are in yellow. These include portions of the left inferior frontal gyrus.

***Stage 1***. In order to identify brain regions consistently associated with spelling across individual subjects we applied an ALE analysis to the set of peaks obtained from the individuals in both control groups. The results revealed a set of significant clusters that represent the locations with the highest likelihood of an activation peak (see Figure 4A; Table 4). The two clusters with the highest ALE values were in the left fusiform gyrus (BA 37) and left inferior frontal gyrus (BA 9/44). Other regions with significant ALE values were all in the left hemisphere and included: left middle frontal gyrus (BA 11), inferior frontal gyrus (BA 47), superior temporal gryus (BA 22), inferior temporal gyrus (BA 37), superior occipital gyrus (BA 19), and putamen.

**Table 4:** **Results of Stage 1 of the IPPC analysis: Subject-based Activation Likelihood Estimates (ALE) for control participants**.

**Location**		**BA**	**Volume (mm^3^)**	**ALE peak (Max/local-max)**			**ALE value**
				***x***	***y***	***z***	
Left	FG	37	30776	−40	−60	−14	7.88
	ITG	37		−44	−44	−16	5.73
	MOG	19		−36	−86	6	5.05
	MTG	22		−50	−36	0	8.17
Left	IFG	9	23288	−42	4	28	7.35
	IFG	44		−50	8	20	5.08
Left	MFG	11	1440	−38	34	−10	4.99
	MFG	11		−28	40	−4	5.3
Left	SOG	19	1192	−30	−70	22	4.71
Left	IFG	47	272	−22	16	−12	4.7
Putamen				−16	16	−8	4.67
Left	STG	22	80	−58	−20	2	4.67

Listed are the peak locations (Talairach) and volumes for clusters with significant ALE values, including both maximum and local-maximum peaks. BA, brodmann area.

Anatomical labels: IFG, inferior frontal gyrus; ITG, inferior temporal gyrus; MOG, middle occipital gyrus; MFG, middle frontal gyrus; SOG, superior occipital gyrus; STG, superior temporal gyrus.

It is worth noting that when DPT's activation map (reported in Figure 3B) is overlaid onto the subject-based ALE map, there is overlap between the control ALE map and DPT's activation map (see Figure 4B) in the left inferior frontal gyrus. This suggests that when taking into account the variability of activation responses across control subjects (as the subject-based ALE analysis does), DPT's pattern of activation may be normal in some regions, and abnormal in others. In the next two Analysis Stages we examine this possibility.

***Stage 2***. First, we identified the set of the peaks from the individual control participants that contributed significantly to each ALE peak identified in Stage 1. We did so by identifying the control participant peaks that were within 2 standard deviations of the UD from each ALE peak or local-maximum peak. The number of control participant peaks that were associated with each ALE peak ranged from 7 to 13. We then characterized the ALE peaks as being either “strong” or “weak” as follows: if at least a majority (≥%50) of the control participants contributed significantly, the ALE peak was categorized as a “strong” normal peak; five ALE peaks were categorized in this manner. If fewer than half of the control participants contributed significantly, the ALE peak was categorized as a “weak” normal peak; seven ALE peaks were categorized in this manner. This distinction between strong and weak ALE peaks served to distinguish between the ALE peaks that represented the most consistent normal neural response for spelling and those that, although significant in the ALE analysis, were less reliably recruited across participants.

**Figure 5:**
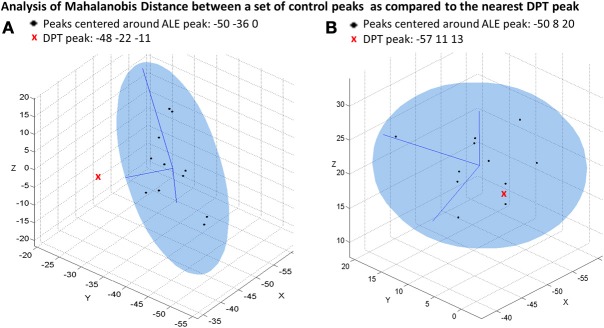
**Mahalanobis distance plots depicting peak activations for DPT and control participants**. The ellipsoids represent the 95% confidence interval of the control data and x, y, and z represent dimensions in Talairach coordinate space. **(A)** Depicts a plot of the individual control participant peaks that contribute to a group ALE peak that is located in the left middle temporal gyrus (centered at −50, −36, 0). This is compared to the nearest DPT peak at (−48, −22, −11); *MD*^2^ = 20.1, *p*-value = 0.0002. **(B)** Depicts a plot corresponding to the control peaks corresponding to a group ALE peak located in the left IFG (centered at −50, 8, 20). This is compared the nearest DPT peak at (−57, 11, 13); *MD*^2^ = 5.3, *p*-value = 0.154.

Second, for each of the 5 peaks associated with the strong normal spelling pattern we then identified the nearest DPT peak (from the list of peaks reported in Table 3) and computed the MD between DPT's peak location and the distribution of the individual control peaks that contributed to the strong group ALE peak (see Table 5). A *p*-value less than 0.05 indicates that the nearest DPT peak was significantly abnormal in relation to the center of the distribution of control peaks contributing to these strong ALE peaks. For the strong ALE peaks identified in the large occipitotemporal cluster (which included portions of the left fusiform gyrus, inferior temporal gyrus, middle occipital gyrus, and middle temporal gyrus) we found that the nearest DPT peaks were outside of the range of each one. This is illustrated in Figure 5A, with the ALE subpeak in the left middle temporal gyrus (centered at −50, −36, 0). The nearest DPT peak was (−48, −22, −11); and a comparison of this location to the set of locations corresponding to the control participant peaks produced an MD^2^ = 20.1 (*p* = 0.0002). As Table 5 indicates, there were no DPT peaks that were normally situated relative to the strong ALE peaks in the left occipitotemporal area. In contrast, as shown in Figure 5B, if we consider the left IFG (BA 44), specifically the IFG ALE peak centered at (−50, 8, 20), we find that DPT had an activation peak located at (−57, 11, 13) that was within normal range (MD^2^ = 5.3, *p* = 0.154).

**Table 5:** **Results of stage 2 of the IPPC analysis**.

**ALE peak locations**							**DPT peaks nearest to to each ALE peak**					**MD comparison**	
**Location**	**BA**	**Peak (Max/local-max))**			**ALE value**	**Control subjects per ALE peak**	**Location**	**BA**	**Peak (Max/local-max)**			***MD***	**Chi-square**
		***x***	***y***	***z***					***x***	***y***	***z***		***p***
FG	37	−40	−60	−14	7.88	13	^*^MTG	21	−48	−22	−11	87.4	<0.001
ITG	37	−44	−44	−16	5.73	12	^*^MTG	21	−48	−22	−11	20.3	<0.001
MTG	22	−50	−36	0	8.17	11	^*^MTG	21	−48	−22	−11	20.1	<0.001
IFG	9	−42	4	28	7.35	13	PreCG	6	−54	2	10	66.4	<0.001
**IFG**	**44**	−**50**	**8**	**20**	**5.08**	**11**	**IFG**	**44**	−**57**	**11**	**13**	**5.3**	**0.154**

“Strong” ALE peaks with contributions from the majority of subjects (≥%50) are reported. Listed for each ALE peak are the number of control participants that contributed to the peak, and its ALE value and location (Talairach). Also reported are the results of Stage 2 of the IPPC Analysis calculating Mahalanobis distances (MD) between each of the significant subject-based ALE peaks and the nearest peak (or subpeak) in DPT's activations. P-values are reported for the chi-square test evaluating the significance of the MDs. Peaks with non-significant MD comparisons are bolded.

* Perilesional peaks (within 5 mm of lesion space); Anatomical Labels: IFG, inferior frontal gyrus; ITG, inferior temporal gyrus.

This analysis reveals that DPT “matches” the most consistently identified “strong normal neural spelling response” pattern in terms of activation in the IFG but not in the occipitotemporal region, in which his lesion is located.

We also evaluated the relationship between DPT's peaks and the “weak” normal peak locations. The results (see Table 6) indicate that, along with the left IFG DPT peak (−57, 11, 13) that was also consistent with the strong normal spelling pattern, there were two DPT peaks that were consistent with weak normal peak locations in the left MTG (−60, −19, −8 and −48, −22, −11).

**Table 6:** **Characterization of the locations of all of DPT's peaks relative to control group peak locations based on stage 2 and 3 analyses**.

**DPT peaks**					**ALE peak locations nearest to each DPT peak**						**MD comparison (Stage 2)**		**PPM comparison (Stage 3)**
**Location BABA**		**Peak**			**Location BA**		**Peak**			**# of controls per ALE peak**	**Chi-square**		**% of control subject PPM values at DPT'speak**
		***x***	***y***	***z***			***x***	***y***	***z***		***MD***	***p*-value**	
**NORMAL: CONSISTENT WITH THE STRONG “NORMAL SPELLING PATTERN”**													
IFG	44	−57	11	13	IFG	44	−50	8	20	11	5.26	0.154	26.3
**NORMAL: CONSISTENT WITH WEAK NORMAL PEAK LOCATIONS**													
^*^MTG	21	−60	−19	−8	STG	22	−58	−20	2	7	6.41	0.093	15.8
^*^MTG	21	−48	−22	−11	STG	22	−58	−20	2	7	6.31	0.097	10.5
**ABNORMAL: SEVERE**													
TP	38	−42	14	−23	IFG	47	−22	16	−12	7	14.1	0.003	10.5
STG	22	−60	−10	4	STG	22	−58	−20	2	7	13.7	0.003	10.5
IFG	45	−54	26	13	IFG	45	−50	8	20	11	22.4	0	10.5
**ABNORMAL: MODERATE**													
STG	22	−51	−7	−11	STG	22	−58	−20	2	7	39	0	15.8
STG	22	−51	11	−5	IFG	44	−50	8	20	11	32.1	0	21.1
IFG	45	−45	20	10	IFG	44	−50	8	20	11	11.2	0.011	15.8
PreCG	6	−54	2	10	IFG	44	−50	8	20	11	8.9	0.031	31.6

Reported are the number of control participants that contributed to each significant control group ALE peak; these values were used to characterize control peak locations as strong/weak (see text for details). Also reported are the results of the calculation of Mahalanobis Distances (MD) between each of DPT's significant peaks and the nearest control group ALE peak. P-values are reported for the chi-square test evaluating the significance of the MDs. On this basis, DPT peaks were categorized as normal/abnormal (see text for details). Reported also, from Stage 3 of the IPPC analysis, are the results of the Peak Probability Map (PPM) comparison, listing the percentage of control participants with a peak within 1 SD of the UD from each of DPT's peaks. The lower the PPM percentage, the more atypical the DPT peak (with fewer nearby control peaks). DPT's non-normal peaks were classified as severely or moderately abnormal (overlapping with fewer than or greater than 11% or normal controls, respectively).

* Perilesional peaks (within 5 mm of lesion space); Anatomical labels: TP, temporal pole; STG, superior temporal gyrus; MTG, middle temporal gyrus; IFG, inferior frontal gyrus; PreCG, precentral gyrus.

While the Stage 2 analysis evaluated whether or not DPT had activation peaks that fell within normally activated regions, it did not consider the *degree* of atypicality of DPT's additional “non-normal” activations. To this end, Stage 3 of the IPPC analysis more closely examines DPT's peaks that were not found to be normal in Stage 2.

***Stage 3***. The final stage of the IPPC analysis involved determining the degree to which DPT's non-normal activation peaks (Table 3) were in locations that were atypical given the range of control participant responses.

**Figure 6:**
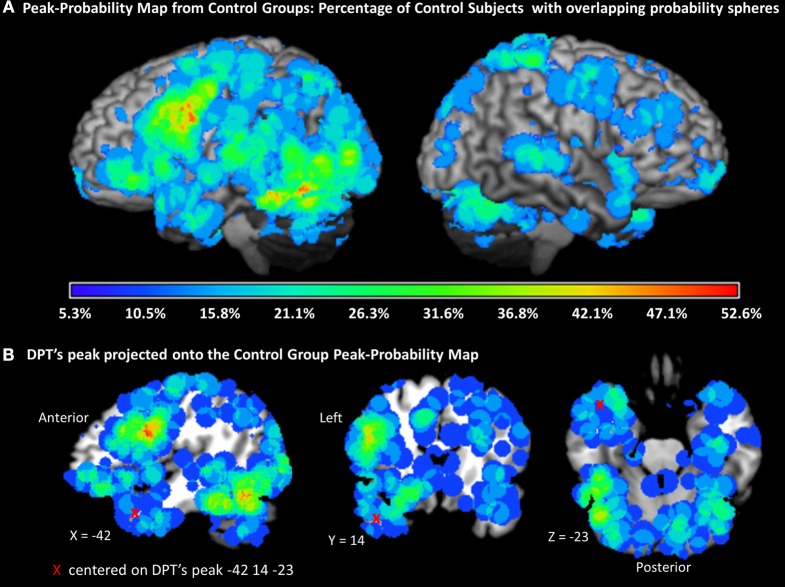
**Peak probability map (PPM)**. **(A)** Map of probability spheres each with a radius of 1 standard deviation of the UD (uncertainty distance), centered on each individual participant control peak and projected onto a standard rendered template brain. The color scale represents the percentage of overlap across all control subjects (total = 19). **(B)** One of DPT's peaks (−42, 14, 23) is represented by a red X. The PPM indicates that the maximum percentage of subjects with probabilistic spheres at the location of DPT's peak is approximately 11%, and therefore that the DPT peak is quite atypical.

To evaluate degree of atypicality, we calculated the percentage of control participants that demonstrated overlapping peak-probability spheres with DPT's peaks. For this analysis, we generated a PPM by generating spheres with a standard deviation of 1 UD radius around each of the control peaks (see Figure 6A). Not surprisingly, the PPM presented in Figure 6A was consistent with the previously reported results from the subject-based ALE analysis in that the regions with the highest percentage of probabilistic sphere overlap across subjects was in the left inferior frontal gyrus and ventral occipitotemporal cortex. We then counted the percentage of control subjects with each of DPT's peaks.

As reported in Table 6, three of the 7 of DPT's peaks that were identified as non-normal in Stage 2 were determined to be in highly atypical locations based on the PPM comparison, i.e., locations where at most 11% of the control subjects also demonstrated high probability of having a significant activation peak. This subset of DPT's peaks is considered to represent the most atypical components of DPT's functional activation pattern. As also indicated in Table 6, DPT's four other non-normal peak locations were considered to be moderately abnormal, overlapping with between 16 and 32% of control participants' peak probability spheres.

### IPPC results summary

**Figure 7:**
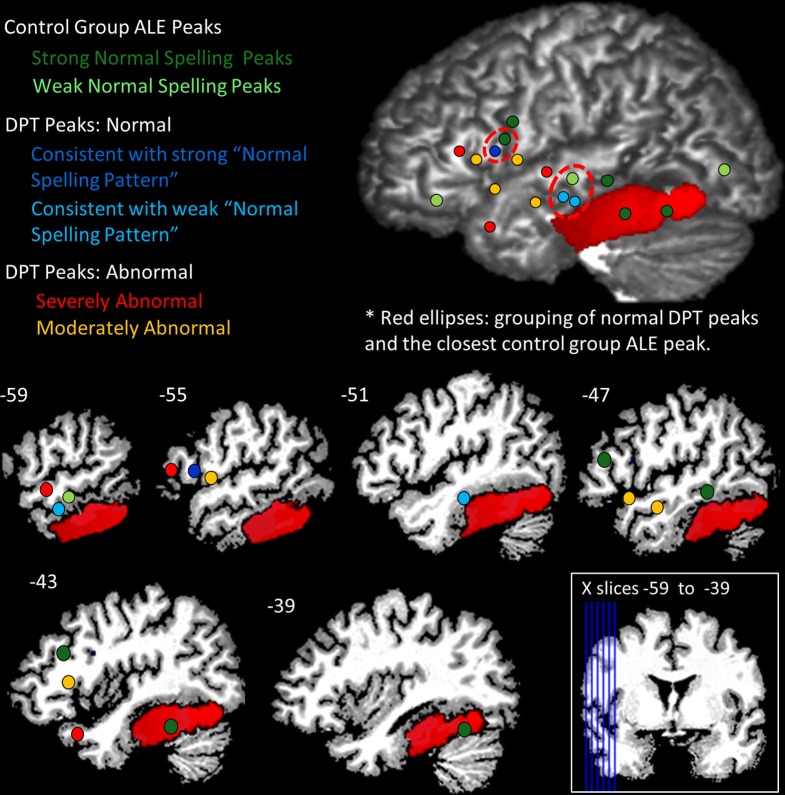
**Summary of IPPC Analysis results**. Each of DPT's peaks and the control group ALE peaks (see Table 5) were projected onto DPT's brain; the red region depicts DPT's lesion. Dots are used to visualize the peak locations and were projected at a depth of 16 mm. Green identifies the location of peaks for the normal group, with dark green depicting “strong” normal activation peaks (the majority of control participants) and light green depicting “weak” normal activation peaks (see text for details). Blue dots are used to depict DPT's activation peaks that were consistent with normal activation peaks: dark blue indicates DPT peak locations consistent with the strong normal activation peaks; light blue depicts DPT's peaks consistent with the weak normal activation peaks. For DPT peaks that were identified as within the normal range, the red dashed circles indicate the grouping of the DPT peaks and the nearest control group peak. Red depicts DPT peaks that were classified as severely abnormal in their location (consistent with fewer than 11% of control participant peaks); orange depicts DPT peaks that were moderately abnormal in their location (consistent with 11–33% of control participant peaks).

The IPPC analysis provides a method for quantitatively evaluating the location of each of DPT's peaks vis a vis the range of control participant activation peak locations. A summary of the results is presented in Table 6 and in Figure 7. Stage 1 analysis of the control participant responses using an ALE approach identified locations with statistically significant likelihoods of corresponding activation peaks. These locations were further characterized as being “strong” or “weak” depending on the proportion of normal participants (>50 vs. <50%) that exhibited peak activations in these locations. In Figure 7, these are depicted in dark and light green, respectively. In Stage 2, Mahalanobis distance analysis was used to characterize each of DPT's peak activation locations relative to the normal peak locations. Figure 7 displays the locations of DPT's peaks that were consistent with strong control group peak locations in dark blue, and those consistent with weak control group peaks in light blue. Stage 3, characterized the degree of abnormality of DPT's non-normal activation peaks using a PPM (probabilistic peak map) approach. DPT's non-normal peaks were characterized as severely or moderately abnormal depending on the degree of overlap (<11 or > 11%) between DPT and normal control participants' peak probability spheres. The results are depicted in Figure 7 with red used to identify the severely abnormal locations and orange the moderately abnormal ones.

## Discussion

In this study we examined the functional activation patterns associated with spelling in DPT, an individual with a lesion to the left occipitotemporal cortex who demonstrated persistent deficits that specifically affected word spelling. Given the important role that this brain region has been shown to play in spelling, it is of considerable interest to understand the brain's functional response (as measured by fMRI) to a lesion in this area. Using a conventional data analysis approach, DPT's fMRI activation pattern for spelling did not overlap with the control group's response pattern. On this basis, his spelling network may have been considered to be entirely abnormal. However, in order to evaluate DPT's activations within the context of the considerable variability in activations across individual participants, we implemented a novel IPPC analysis that allows for a comparison of the locations of activation peaks in one individual to activation peak locations observed in a set of individuals. Briefly, Stage 1 of the IPPC analysis involves a new application of the ALE analysis technique (Turkeltaub et al., 2002) that provided a probabilistic approach for combining data across a set of control participants to characterize the “normal neural response to spelling.” Stage 2 of the IPPC analysis uses the MD measure to identify activation peaks that are consistent with the “normal” group-based network and activation peaks that are not consistent with the normal group pattern. Stage 3 of the IPPC analysis uses a Peak Probability Map approach to characterize the degree of abnormality of the “non-normal” activation peaks.

When IPPC was applied we found that DPT demonstrated both normal and abnormal loci of activation, results which are summarized in Figure 7. There were three main findings. First, DPT exhibited activation in a key area within the normal neural spelling network, specifically in the left inferior frontal gyrus (BA 45) (as denoted by the dark blue dot in Figure 7). This indicates that at least some of the normal spelling network remained intact. The second finding concerns perilesional cortex. DPT exhibited normal perilesional activation largely anterior to his lesion in the left middle temporal gyrus (BA 21) (as denoted by the light blue dots in Figure 7). This may reflect restructuring of remaining, fragmentary fusiform/inferior temporal gyrus networks that are normally available for spelling. Third, DPT demonstrated activation in atypical regions for spelling including the left anterior temporal pole of the STG (BA 38), left inferior frontal gyrus (BA 45), and left superior temporal gyrus (BA 22) (as denoted by the red dots in Figure 7); these areas may contribute to compensation for the lack of functional use of the left occipitotemporal cortex. Overall, these results reveal that damage to the left ventral occipitotemporal cortex that impacts word spelling can lead to functional reorganization within the left hemisphere that calls upon both “old,” “new” and perilesional regions of the left temporal and frontal lobes.

### The normal neural pattern for spelling

We identified the normal neural response to spelling by applying the ALE analysis to fMRI spelling data from a set of control subjects. To date this is the first application of the ALE analysis technique to a set of individual participants (treating each as an *n* = 1 study) with the aim of identifying cross-participant locations of high cluster-peak probabilities. The results of this subject-based ALE analysis revealed that the two areas most consistently identified across the sets of control participants were in the left ventral occipitotemporal cortex and the left inferior frontal junction regions. These two regions are also the most consistently identified in a meta-analysis of neuroimaging studies of spelling (Purcell et al., 2011) see also (Planton et al., 2013). This correspondence of findings provided support for this novel extension of the ALE analysis and also provided the basis for implementing the IPPC analysis that allowed us to evaluate whether or not DPT's functional activation patterns deviated from the normal pattern in terms of the locations of likely activation peaks.

### Normal spelling activation within a restructured spelling network

One of the main findings of the IPPC analysis was that DPT demonstrated activation that could clearly be considered to be normal. Specifically, a cluster peak in the left IFG (−57, 11, 13; BA 44) was within the normal range of peak activation locations for the most highly consistent normal pattern for spelling as reported in Table 5, indicating that at least some of DPT's normal spelling network was intact and engaged in spelling.

Furthermore, we also examined other spelling areas reported in Table 6 that, although not the most consistently recruited across the control participants, were still measurably relevant to the spelling network in the subject-based ALE analysis (denoted by the light green spheres in Figure 7). This analysis revealed that DPT had two activation peaks that were within range of one of these “weak” normal activation sites, namely in the left middle temporal gyrus (BA 21). These findings underscore the fact that multiple components of DPT's activation pattern in spelling were consistent with the patterns generated by many control participants.

Critically, these findings were not identified by overlaying the functional maps from DPT control group average results (Figure 3), whereas there was observed overlap between DPT and the subject-based ALE results, i.e., when the variability in control subject responses is taken into consideration. This finding emphasizes the ability of the IPPC analysis method to determine not only what areas of functional activation are abnormally situated, but also, which are in normal locations.

### Perilesional recruitment

The region of the left mid-fusiform gyrus and the lateral occipitotemporal sulcus (bordering the ITG) is one of the areas most consistently identified across studies of spelling (Purcell et al., 2011), and has long been associated with orthographic lexical processing for reading (Kawahata et al., 1988; Cohen et al., 2003; Hillis et al., 2005; Gaillard et al., 2006) and more recently with spelling (Rapcsak and Beeson, 2004; Philipose et al., 2007; Tsapkini and Rapp, 2010). DPT's resection largely removed this region. However, the analyses revealed activations that abutted this area in perilesional cortex. Specifically, activations associated with peaks in MTG (−60, −19, −8 and −48, −22, −11) were located perilesionally. This finding suggests the recruitment and possible restructuring of areas near the lesion that may have retained fragmentary aspects of the original spelling networks in this region (see Tsapkini et al., 2011).

### Functional reorganization subsequent to damage to the spelling network

Another key finding of the IPPC analysis was that DPT exhibited activation peaks in the left temporal pole, superior temporal gyrus, and the left inferior frontal gyrus that were clearly atypical relative to the control group and, therefore, indicative of novel functional recruitment.

With respect to the left temporal pole, it is interesting to note that recruitment of this region is consistent with findings from previous research with DPT that identified the anterior temporal lobes (bilaterally) with functional reorganization for reading (Tsapkini et al., 2011). A possible interpretation for the recruitment of this specific area is that the left temporal pole is typically called upon to spell certain classes of words and that when the spelling network is damaged; it is recruited to a greater degree. This is supported by recent work that found that atrophy to the left temporal pole, along with the fusiform gyrus, was associated with deficits in exception word spelling (Shim et al., 2012). One possible role of the left anterior temporal pole in spelling is the mapping of semantic information with orthographic representations. This is supported by numerous studies which have identified the bilateral temporal pole as being generally associated with semantic processing. For instance, atrophy to the temporal pole is associated with impaired ability to access the meaning of words in semantic dementia (Ralph et al., 2003; Woollams et al., 2007). Also, activation in fMRI tasks involving semantic processing tend to activate this region (Visser et al., 2010), and a study involving lesion symptom mapping specifically relevant to semantic processing has also implicated the anterior temporal lobe as being important for semantics across a group of aphasiac patients (Schwartz et al., 2009). Interestingly, in work that actually implemented semantic training in an acquired dyslexic individual, treatment-related neural changes were observed in the left temporal pole (Kurland et al., 2008). With regard to acquired dysgraphia, the cognitive profile of DPT indicates that his ability to represent semantic information was intact, but that there was a specific deficit in gaining access to orthographic word forms from meaning in spelling (Tsapkini and Rapp, 2010; Tsapkini et al., 2011). Therefore, the left anterior temporal lobe activation may be associated with a compensatory semantically driven response that occurs when there is difficulty retrieving orthographic representations from their meanings.

The specific cognitive mechanism associated with the atypical activation identified in the left inferior frontal gyrus (BA 45) is difficult to determine due to the wide variety of cognitive functions that BA 45 has been associated with e.g. (Liakakis et al., 2011). For instance, one possibility is that this activation may be associated with compensatory phonological processing. This is supported by numerous studies which have found this portion of the left IFG to be important for normal phonological processing reading, i.e., the mapping of correct orthographic units to their phonological counterparts e.g. (Pugh et al., 2001; Fiez et al., 2006; Taylor et al., 2013). With regards to spelling, recent work has found that for spelling this area may be involved in correctly matching sounds to letters. This is supported by the work of Shim et al. (2012) who reported that errors in spelling that were phonetically implausible were correlated with thinning in the left IFG (BA 44/45). For DPT, it is possible that although in some area of the left IFG (BA 45) there is normal recruitment of the spelling network, the damage to the network in the occipitotemporal region triggered more extensive utilization of BA 45 when spelling. However, due to the numerous proposed functional roles of the left IFG, other explanations are certainly possible.

The abnormal activation in the STG (BA 22) is very plausibly related to greater than normal reliance on sublexical phoneme-to-grapheme conversion mechanisms. These mechanisms have been linked to peri-sylvian regions in the STG/STS/MTG region in neuroimaging studies (Booth et al., 2002, 2003) as well as in neuropsychological studies (Rapcsak et al., 2009), with the latter showing a strong relationship between lesions to this area and deficits that specifically affect the ability to convert phonological strings to orthographic strings for novel words or pseudowords. In this regard, it is noteworthy that DPT was completely normal in his ability to spell pseudowords and that his errors in word spelling were limited to words with irregular, unpredictable spellings (e.g., TYPE) for which he produced only phonologically plausible errors (e.g., TIPE). This pattern of performance is specifically diagnostic of over-reliance on phoneme-to-grapheme conversion, providing a clear motivation for unusual levels of recruitment of areas associated with these processes.

### IPPC—focus on the neuro-topography of peak activations rather than cluster volumes

In order to characterize the functional neuro-topography of a brain lesioned individual, the IPPC approach analyzes peak locations of activation rather than the magnitude or volume of activation clusters. The IPPC analysis focuses on peak locations for the following reasons. First, the specification of peak locations is the most common method of characterizing cluster locations in fMRI investigations and is almost universally used to characterize the neuro-topography of a given cognitive process (e.g., process X recruits regions, A, B, and C). Second, the size a cluster is highly dependent on the significance thresholds that are used. If you examine individual subject data (for any study) you find that, at any given threshold, there is typically enormous variability in cluster sizes across normal participants. Ideally we would want to use a fixed threshold across individuals that would yield a set of clusters that are discrete and representative of distinct functional brain regions. However, there is no work determining what the appropriate thresholds should be in order to yield such results and, it would not be surprising, if these would turn out to be different for different cognitive functions and brain areas. Focusing on characterizing the neuro-topography of peak activations allows one to circumvent many of the complications associated with cluster volume, allowing one to directly address questions concerning the comparison of normal vs. abnormal activation topographies.

## Conclusions

This research provides information regarding the manner by which the network of regions associated with spelling responds when the left ventral occipitotemporal cortex is damaged. Overall, the findings indicate that damage to the left ventral occipiotemporal cortex is associated with ipsilesional activation for spelling. The ipsilesional responses include recruitment of perilesional sites, as well as sites within the normal spelling network and novel brain areas. In general, the findings are consistent with the proposal by Turkeltaub et al. (2011) that lesions to non-IFG areas tend to be associated with ipsilesional patterns of activation. Furthermore, this work illustrates that the complex functional response to a lesion affecting a critical component of the spelling network requires an analysis method that can distinguish between the normal vs. abnormal functional responses. The IPPC analysis method provides a valuable tool for characterizing functional responses in neurologically impaired individuals and, in fact, can be applied more broadly to other situations that require comparing functional neuroimaging data from a single individual with data from a set of individuals.

### Conflict of interest statement

The authors declare that the research was conducted in the absence of any commercial or financial relationships that could be construed as a potential conflict of interest.

## Footnotes

^1^In Eickhoff et al. (2009) an fMRI study of finger tapping was used to estimate variance of individual subject peaks across subjects and across different analysis methods (see Eickhoff et al., 2009). From these variance estimates measures they calculated the appropriate FWHM of a Gaussian distribution which reflects the location uncertainty of a peak location in fMRI. Two different FWHM measures were obtained: one which reflects the peak location variability across subjects (FWHMsub) and another which reflects the peak location variability across different normalization templates (FWHMtemp). Based on the empirical findings of Eickhoff et al. (2009) the FWHMsub was estimated to be 17.2 mm and the FWHMtemp was estimated to be 8.41. Based on the equations 2–4 in Eickhoff et al. (2009) we estimated the standard deviation of the combined FWHM employed in Eickhoff et al. (2009), referred to as the standard deviation of the uncertainty distance (UD); this was done via the following equation: *SDud* = √[(*FWHMsub*/√*N*)^∧2^ + *FWHMtemp*^∧2^)/√(8 × log(2)]. For this work N refers the number of subjects per set of peaks; N is 1 for the purposes of this work because we are treating each individual subject as separate data sets.

^2^This was done by first estimating the center, *c*, and covariance matrix, *S*, of the controls' peak locations and then calculating the squared distance using the standard equation MD2 = (*x* − *c*)^*T*^ × *S*^−1^ × (*x* − *c*).
